# Leukemia Inhibitory Factor (LIF) Inhibition during Mid-Gestation Impairs Trophoblast Invasion and Spiral Artery Remodelling during Pregnancy in Mice

**DOI:** 10.1371/journal.pone.0129110

**Published:** 2015-10-19

**Authors:** Amy Winship, Jeanne Correia, Jian-Guo Zhang, Nicos A. Nicola, Evdokia Dimitriadis

**Affiliations:** 1 The Hudson Institute, Melbourne, Australia; 2 Department of Anatomy and Developmental Biology, Monash University, Melbourne, Australia; 3 The Walter and Eliza Hall Institute of Medical Research, Melbourne, Australia; 4 Department of Medical Biology, The University of Melbourne, Melbourne, Australia; Chinese Academy of Sciences, CHINA

## Abstract

The placenta forms the interface between the maternal and fetal circulation and is critical for the establishment of a healthy pregnancy. Trophoblast cell proliferation, migration and invasion into the endometrium are fundamental events in the initiation of placentation. Leukemia inhibitory factor (LIF) has been shown to promote trophoblast invasion *in vitro*, however its precise role in trophoblast invasion *in vivo* is unknown. We hypothesized that LIF would be required for normal trophoblast invasion and spiral artery remodeling in mice. Both LIF and its receptor (LIFRα) co-localized with cytokeratin-positive invasive endovascular extravillous trophoblasts (EVT) in mouse implantation sites during mid-gestation. Temporally blocking LIF action during specific periods of placental development via administration of our unique LIFRα antagonist, PEGLA, resulted in abnormal trophoblast invasion and impaired spiral artery remodeling compared to PEG control. PEGLA-treated mouse decidual vessels were characterized by retention of α-smooth muscle actin (αSMA)-positive vascular smooth muscle cells (VSMCs), while PEG control decidual vessels were remodelled by cytokeratin-positive trophoblasts. LIF blockade did not alter F4/80-positive decidual macrophage numbers between treatment groups, but resulted in down-regulation of decidual transcript levels of monocyte chemoattractant protein-1 (*MCP-1*) and interleukin-10 (*IL-10*), which are important immune cell activation factors that promote spiral artery remodeling during pregnancy. Our data suggest that LIF plays an important role in trophoblast invasion *in vivo* and may facilitate trophoblast-decidual-immune cell cross talk to enable adequate spiral artery remodeling.

## Introduction

Trophoblast cell proliferation, migration and invasion into the endometrium are critical events in the initiation of placental development [1]. Specialized extravillous trophoblast (EVT) cells migrate and invade into the decidua and remodel the maternal spiral arterioles to create wide-bore arteries and reduce utero-placental resistance [2]. Inadequate or inappropriate spiral artery remodeling and placentation is thought to be a leading cause of pregnancy complications [3].

Placental development is highly regulated spatially and temporally by numerous factors, such as cytokines produced within the local uterine environment [4]. Such factors can ultimately determine the success or failure of pregnancy. Leukemia inhibitory factor (LIF) is one of the most important cytokines in the female reproductive tract [5]. LIF is a member of the Interleukin-6 (IL-6) family of cytokines and is a secreted glycoprotein that signals via the LIF receptor α-chain (LIFRα)/gp130 heterodimeric complex to activate the downstream signalling cascade including the JAK-STAT [1, 6–9] and the extracellular signal regulated kinase (ERK) pathways [10].

LIF is required for uterine blastocyst implantation in mice [11, 12] and is thought to play a critical role in implantation in primates and women [13–15]. LIF-null female mice are infertile due to defects in embryo implantation [14, 16]. Since implantation fails, these mice are not useful in investigating the role of LIF in placentation or trophoblast function. We have shown that blocking endogenous LIF action during the peri-implantation period using a novel polyethylene glycol (PEG) conjugated LIF-antagonist (PEGLA) mimicked this phenotype [12, 17]. Conversely, LIFR-knockout mice are perinatal lethal and die within 24 hours of birth [18]. In these mice, placental morphology is dramatically altered, which likely contributes to the perinatal loss. However the fetus is also LIFR-deficient, which results in impaired skeletal and neuronal development [18], preventing the generation of adult LIFR^-/-^ mice to investigate the role of LIF in placentation. These studies do highlight however, the critical importance of LIF action during pregnancy.

Well-characterised animal models can provide insight into the mechanisms of abnormal placentation. The mouse and human placenta share a high degree of proteomic and molecular genetic homology [19] and overall physiological similarities [20]. EVT remodeling of maternal spiral arteries occurs in both humans and mice, although this remodeling is shallower in mice [21]. LIF and LIFR have never been localized in the mouse placenta, so it is unclear in which cell types LIF signalling is most important. In women, *LIF* and *LIFR* mRNA and LIFR protein are expressed in the chorionic villi, decidua and EVTs of first trimester and term placenta/decidua [22, 23]. Functionally, LIF activates STAT3 in human primary extravillous trophoblast (EVT) cells and stimulates their adhesion to primary endometrial epithelial cells [24]. LIF also promotes primary human first trimester placental villous trophoblast migration and invasion [25], suggesting a potential role in EVT invasion and spiral artery remodeling, however, this has never been investigated *in vivo*.

Strong expression of *LIF* mRNA has been also detected in decidual leukocytes in women [22], suggesting that LIF may mediate interactions between maternal decidual leukocytes and invading trophoblasts. During optimal spiral artery remodeling, there is a transient influx of decidual leukocytes, including activated macrophages, thought to initiate the disruption of the organized VSMC layer and endothelium required for trophoblast penetration of the vessel [26]. Impaired macrophage recruitment or activation can prevent complete spiral artery remodeling [27]. However, it is unknown whether LIF alters vessel remodeling and whether this occurs via a macrophage-dependent mechanism.

Due to the pattern of localisation of LIF in human EVTs and its functional in primary human first trimester EVT *in vitro*, we hypothesised that LIF is required for normal trophoblast invasion and spiral artery remodelling at the fetal-maternal interface *in vivo*. Given the difficulties of studying first trimester placental development in women, we employed a mouse model and co-localized LIF and LIFR with cytokeratin-positive trophoblasts in wild-type mouse decidua. In order to circumvent the lack of available knockout mouse models, we utilized our unique LIF inhibitor, PEGLA to determine the effect of transient LIF blockade during trophoblast invasion and spiral artery remodeling in mice.

## Materials and Methods

### Ethics statement

All procedures were approved by the Monash Medical Centre Animal Ethics Committee (#MMCB/2012/17) and followed the NHMRC Australian Code of Practice for the Care and Use of Animals for Scientific Purposes (S1 Table).

### Wild-type mouse implantation site tissue collection

Female (virgin 8–12 weeks old) and male C57BL/ 6J mice (Monash Animal Services, Clayton, Australia) were housed under conventional conditions, with food and water available *ad libitum* and held in a 12h light and dark cycle. Implantation sites were collected from wild-type mated female mice on embryonic days (E) 6–17 of gestation (E0 = day of plug detection) (n = 3 mice/time point). Whole, intact implantation sites (IS) containing the placenta, maternal decidua and fetus were dissected out and fixed in 10% neutral buffered formalin overnight, or snap frozen.

### LIF inhibition during pregnancy in mice

We produced polyethylene glycol (PEG) conjugated LIF-antagonist (PEGLA) as previously described [12]. The LIF antagonist binds to the LIF receptor α-chain with high affinity but no longer binds to the co-signal transducing subunit, gp130, preventing the initiation of downstream signalling. LA was covalently conjugated to PEG (PEGLA) to increase the period of serum retention [28]. To inhibit LIF action, mated female mice were administered by intraperitoneal (IP) injection with 600μg/dose PEGLA (20mg/kg/dose) or equal molarity PEG as a control (as optimized previously [17, 29, 30]), twice daily at E8-10 or E10-13 (n = 4 mice/group). Mice were killed at E10 or E13 of pregnancy respectively, on the final day of PEGLA treatment. Whole, intact implantation sites (IS) containing the placenta, maternal decidua and fetus were dissected out and fixed in 10% neutral buffered formalin overnight, or snap frozen.

### RNA extraction and polymerase chain reaction

Total RNA was isolated from snap frozen wild-type or PEG/PEGLA treated mouse decidual tissue using TriReagent. Genomic DNA was digested using the DNAfree kit (Ambion) according to the manufacturer’s instructions. RNA samples were analysed by spectrophotometry (Nanodrop) at an absorbance ratio of A260/280nm to determine RNA concentration, yield and purity. cDNA was synthesized from total RNA (250ng) using Superscript III reverse transcriptase (Invitrogen) and analyzed by spectrophotometry at an absorbance ratio of A260/280nm to determine concentration and purity. PCR reactions were performed using PCR express machine (Thermo Fisher Scientific) and GoTaq master mix (Promega) according to the manufacturer’s instructions. cDNA was analysed for LIF, LIFR, gp130, MCP-1, IL-10, IFN-γ and β2-microglobulin (Table 1) using reaction conditions of an initial denaturation at 95°C for 3 mins, followed by 30 cycles of: denaturation, 95°C for 30 secs; annealing, 60°C for 30 secs; extension, 72°C for 1 min; with a final extension at 72°C for 10 min. The PCR products were run on a 1.5% agarose gel with 1000bp DNA ladder (Invitrogen). To semi-quantify gene expression, the gel was scanned using Bio-Rad Gel Doc EZ Imager and band intensity (optical density) of the gene of interest was normalized to β2-microglobulin, using Image lab Software (version 4.1, Bio-Rad).

**Table 1:** Primer sequences.

Primer	Sequence: forward (F) and reverse (R)
LIF	F 5’-TGAACCAGATCAGGAGCCT-3’
	R 5’-CCACATAGCTTGTCCAGGTTGTT-3’
LIFR	F 5’-GTGGCAGTGGCTGTCATTGTTGGAGTGGT-3’
	R 5’-TCATCTGCGGCTGGGTTTGGTATTTCTTC-3’
GP130	F 5’-CATAGTCGTGCCTGTGTGCT-3’
	R 5’-GCCGTCCGAGTACATTTGAT-3’
MCP-1	F 5’- AGCACCAGCCAACTCTCACT -3’
	R 5’- TCATTGGGATCATCTTGCTG -3’
IL-10	F 5’- TGGCATGAGGATCAGCAGGG -3’
	R 5’- GGCAGTCCGCAGCTCTAGG -3’
IFN-γ	F 5’- GCGTCATTGAATCACACCTG -3’
	R 5’- TGAGCTCATTGAATGCTTGG -3’
β-2M	F 5’-GGTCTTTCTGGTGCTTGTCTCA-3’
	R 5’-GTTCGGCTTCCCATTCTCC-3’

### Immunohistochemistry and staining analysis

Serial sections (2μm) were obtained from paraffin embedded mouse implantation sites containing decidua (n = 3/time point) at mid-gestation (E10 and E13). Paraffin embedded PEG or PEGLA treated mouse implantation sites (n = 4/treatment group) were sectioned (5μm). All tissue sections were dewaxed in histosol and rehydrated in a graded series of ethanol. For immunohistochemistry, sections were microwaved at high power (700W) in 0.01M sodium citrate (pH 6.0) for 5min. Endogenous peroxidase activity was quenched with 3% H_2_O_2_ in methanol for 10min and tissues incubated with non-immune blocking solution (10% normal goat serum, 2% normal mouse serum) diluted in 1×Tris-buffered saline (TBS) for 30min. Primary antibody for LIFRα (1:100; R&D Systems #AF-249NA), LIF (1:100; R&D Systems #AF-250NA), pan-cytokeratin (1:200; Santa Cruz #H-240), α-SMA (1:200; Dako #M085129), F4/80 (1:200; Serotec) or isotype negative control goat IgG in blocking solution were applied for 18h incubated at 4°C. After stringent washing with 0.6% Tween-20 in TBS, antibody localisation was detected by sequential application of biotinylated horse anti-goat IgG (1:200; Vector Laboratories) in blocking solution for 30 min and an avidin-biotin complex conjugated to HRP (Vector Laboratories). Protein was visualized as a brown precipitate using diaminobenzidine tetrahydrochloride substrate (Dako). Sections were counterstained with Harris hematoxylin (Sigma Chemicals), and mounted. For immunofluorescence, formalin-fixed sections were treated as described above except: non-immune serum diluted in and washes performed in phosphate buffered saline; primary antibody for pan-cytokeratin (1:100), α-SMA (1:100) or non-immune goat IgG (isotype negative control); secondary antibody incubation (Donkey α-mouse alexa fluor 488 and Donkey α-goat alexa fluor 594; both 1:200) in non-immune serum for 2h at room temperature; following further washes, sections were mounted using Vectastain containing DAPI (DAKO). Digital photographs at 1X were taken and CellSense software was used to quantify pixel density and expressed as intensity per area to give a percentage. Decidual area was quantified by measuring the cross-sectional area and expressed per total implantation site area as a percentage.

### Statistical analysis

GraphPad Prism 6.0 (GraphPad Software) was used for all statistical analyses. Data were analysed by students t-test when comparing two groups, or ANOVA followed by Tukey’s post-hoc test when comparing more than two groups. Data were expressed as the mean ± SEM. Differences were considered significant at p<0.05.

## Results

### *LIF*, *LIFR* and *gp130* are expressed in the mouse decidua throughout gestation

**Fig 1:**
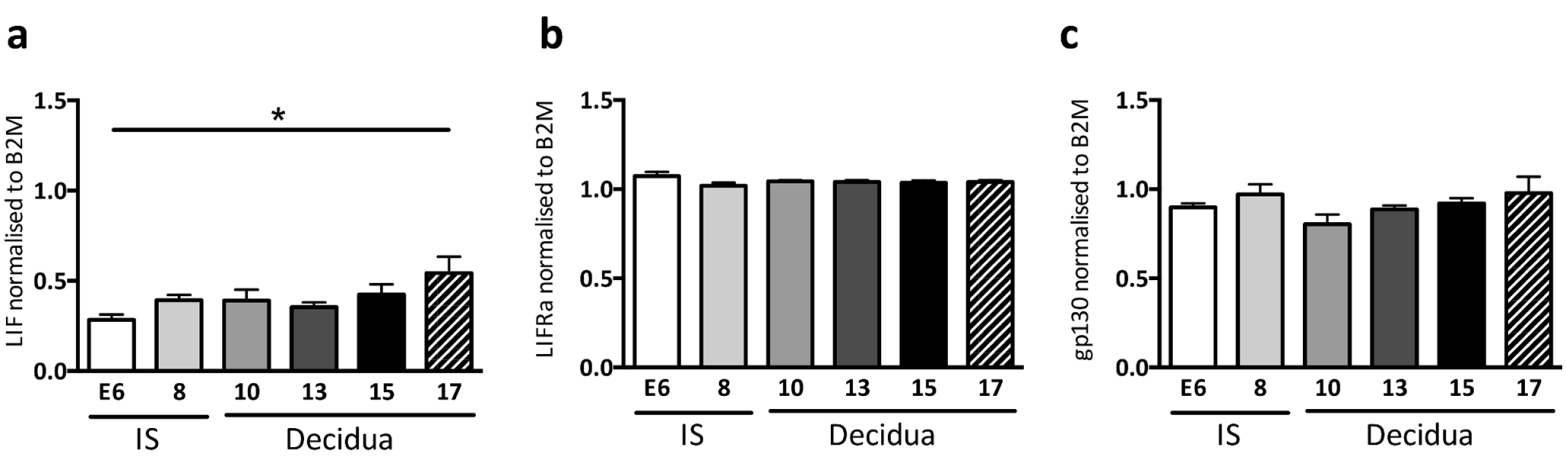
LIF and LIFRα mRNA expression levels in wild-type mouse decidua throughout gestation. Wild type (WT) mouse implantation sites were collected from n = 3 mice/time point. E6, 8 and 10 embryos were analyzed as whole implantation sites (IS) and E13, 15 and 17 were dissected to obtain the decidua only. **(a)** LIF, **(b)** LIFRα and **(c)** gp130 mRNA expression was determined by semi-quantitative PCR normalized to β2-microglobulin. Data are mean ± SEM, ANOVA, Tukey’s post-hoc test, *p<0.05, n = 3/time point.

*LIF* mRNA was produced in the mouse decidua throughout gestation and levels were significantly increased in E17 late-gestation decidua compared to E6 whole implantation sites (Fig 1). Levels of *LIFR* and *gp130* mRNA were unchanged in whole mouse implantation sites at E6 and E8, or decidua alone from E10-17 of gestation in mice (Fig 1).

### LIF and LIFR co-localize with endovascular EVTs in mouse implantation sites

**Fig 2:**
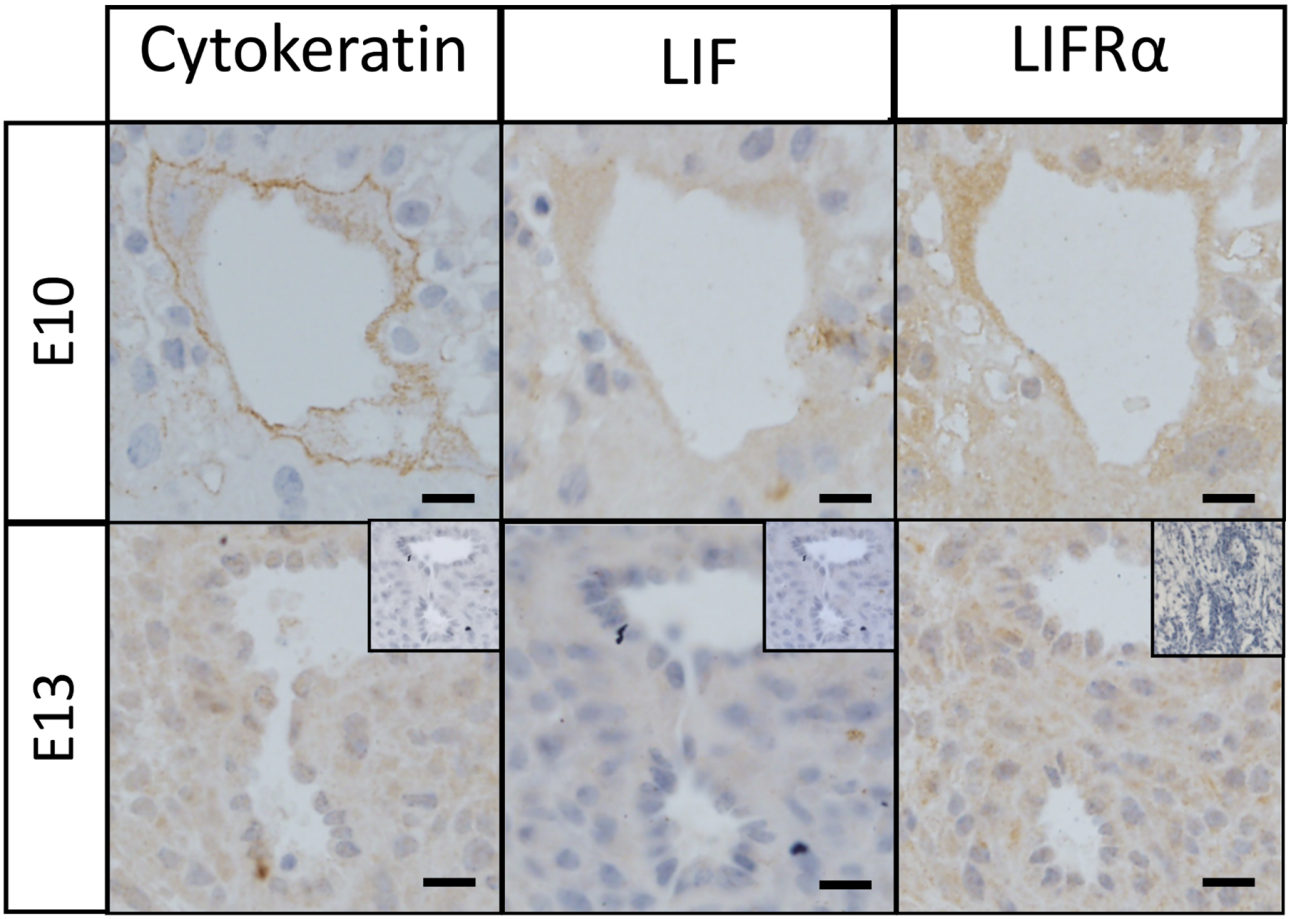
LIF and LIFRα co-localization with cytokeratin to detect invasive EVTs in wild-type mouse decidua. Wild type (WT) mouse implantation sites were collected from n = 3 mice/time point and 2μm serial sections were immunostained for cytokeratin, LIF and LIFRα. Representative photomicrographs of mid-gestation (E10 and E13) implantation site sections are shown here. Both LIF and LIFRα co-localized with cytokeratin in maternal decidual vessels. Bars represent 20μm. Insets are negative controls.

To determine whether EVTs in mice produce LIF and LIFR, we co-localized these with cytokeratin-positive trophoblasts in serial sections from wild-type implantation sites at mid-gestation (E13). Both LIF and LIFR localized to endovascular EVTs in mouse implantation sites (Fig 2).

### LIF blockade during placentation impairs trophoblast invasion and spiral artery remodeling in mice

**Fig 3:**
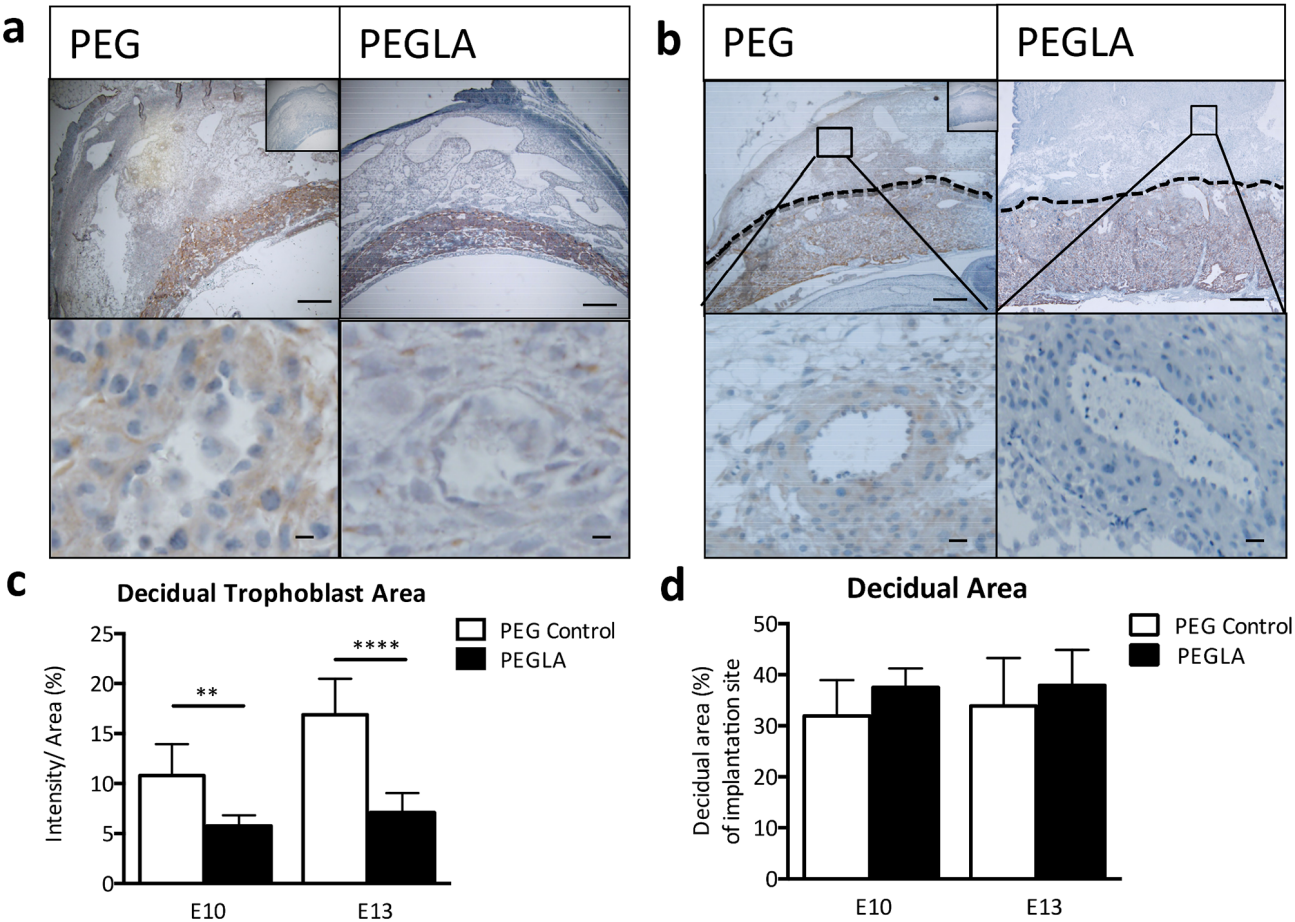
LIF inhibition during placental development reduced invasive decidual trophoblast area in mice. Cytokeratin immunostaining was performed on **(a)** E10 or **(b)** E13 implantation sites treated with PEG control or PEGLA from E8-10 or E10-13 respectively. Bars represent 200μm (upper panel) and 20μm (lower panel). Insets are negative controls. **(c)** Staining intensity was analysed in 3 mid-sagital implantation site sections per mouse (n = 4/group) and averaged using CellSens software, quantified as staining intensity (pixels) per decidual area (%) (highlighted in **(b)**; dotted line). Decidual area was quantified as % area of the whole implantation site (highlighted in **(b)**; dotted line). Data are mean ± SEM, students t-test, **p<0.01, ****p<0.0001.

**Fig 4:**
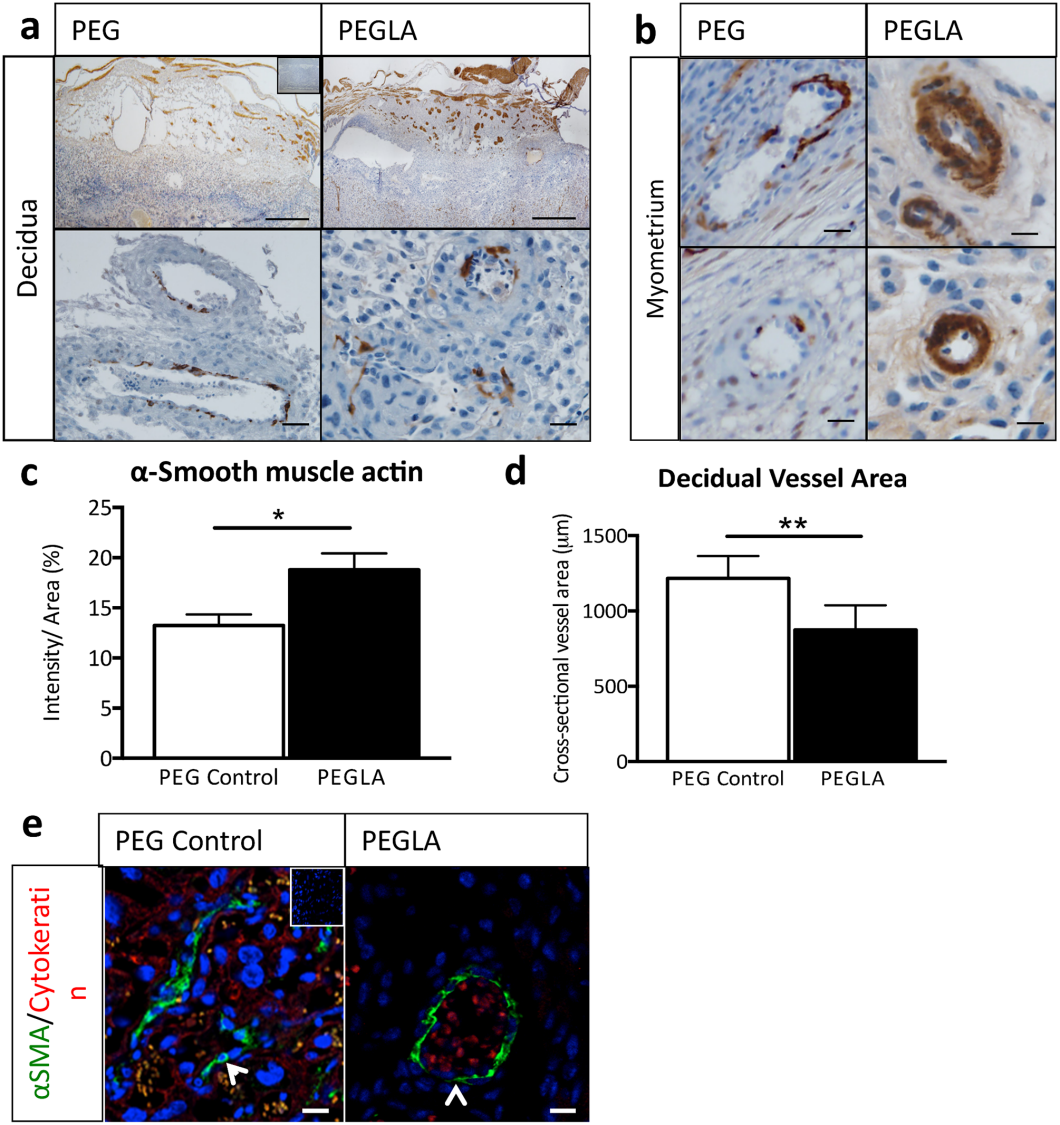
LIF inhibition during placental development impairs spiral artery remodelling in mice. **(a, b)** Representative photomicrographs of E13 implantation sites treated with PEG control or PEGLA from E10-13, immunostained for α-smooth muscle actin (SMA). Bars represent 1mm (**(a)** top panel), 50μm (**(a)** lower panel) and 20μm **(b)**. Inset is the negative control. Decidual **(a)** and myometrial **(b)** vessels from PEGLA treated pregnant mice have altered, narrow vessel morphology and thicker smooth muscle lining compared to PEG control. **(c)** Staining intensity was analysed in 3 mid-sagital implantation site sections per mouse (n = 4/group) and averaged using CellSens software, quantified as staining intensity (pixels) per decidual area (%). **(d)** Cross-sectional decidual vessel area (μm) was measured using CellSens software. Data are mean ± SEM, students t-test, *p<0.05, **p<0.01. **(e)** Cytokeratin positive (red) trophoblast and α-SMA positive (green) smooth muscle cells (arrows) were co-localized using immunofluorescence. Inset is the negative control.

In order to determine the role of LIF on trophoblast invasion *in vivo*, which occurs maximally during mid-gestation from E8-E13 [31], mice were treated with PEG vehicle control or PEGLA from either E8-10 or E10-13 to block LIF action. Mice were sacrificed on the final day of PEGLA treatment. Whole implantation site sections were immunostained with cytokeratin to detect invasive trophoblasts at the fetal-maternal interface at E10 (Fig 3) and E13 (Fig 3). Quantification of this immunostaining at showed a significant reduction in decidual trophoblast area in PEGLA treated mice at E10 (5.77% ± 1.05 vs. PEG control, 10.80% ± 1.11; n = 4/gp, p<0.01) and at also E13 (PEGLA, 6.99% ± 0.69 vs. PEG control, 16.88% ± 1.18; n = 4/gp, p<0.0001) (Fig 3). While decidual area was unchanged between treatment groups (Fig 3). Additionally, at E13 there was a significant increase in decidual and myometrial α-SMA area in PEGLA treated mice (18.79% ± 1.63 vs. PEG control, 13.25% ± 1.07; n = 4/gp, p<0.05; Fig 4a). This increase was evident morphologically from a thicker α-SMA-positive VSMC lining in both decidual and myometrial maternal arteries (Fig 4). Consequently, cross-sectional decidual vessel area was significantly reduced in PEGLA treated mice (874μm ± 67 vs. PEG control, 1216μm ± 51; n = 4/gp, p<0.01) (Fig 4). Immunofluorescence co-localization of cytokeratin with α-SMA at E13 demonstrated that trophoblasts had not successfully displaced the VSMC lining of decidual vessels in PEGLA treated mice, to the same extent as PEG control mice (Fig 4).

### LIF inhibition reduced decidual transcript levels of immune cell activation factors that promote spiral artery remodeling during pregnancy

**Fig 5:**
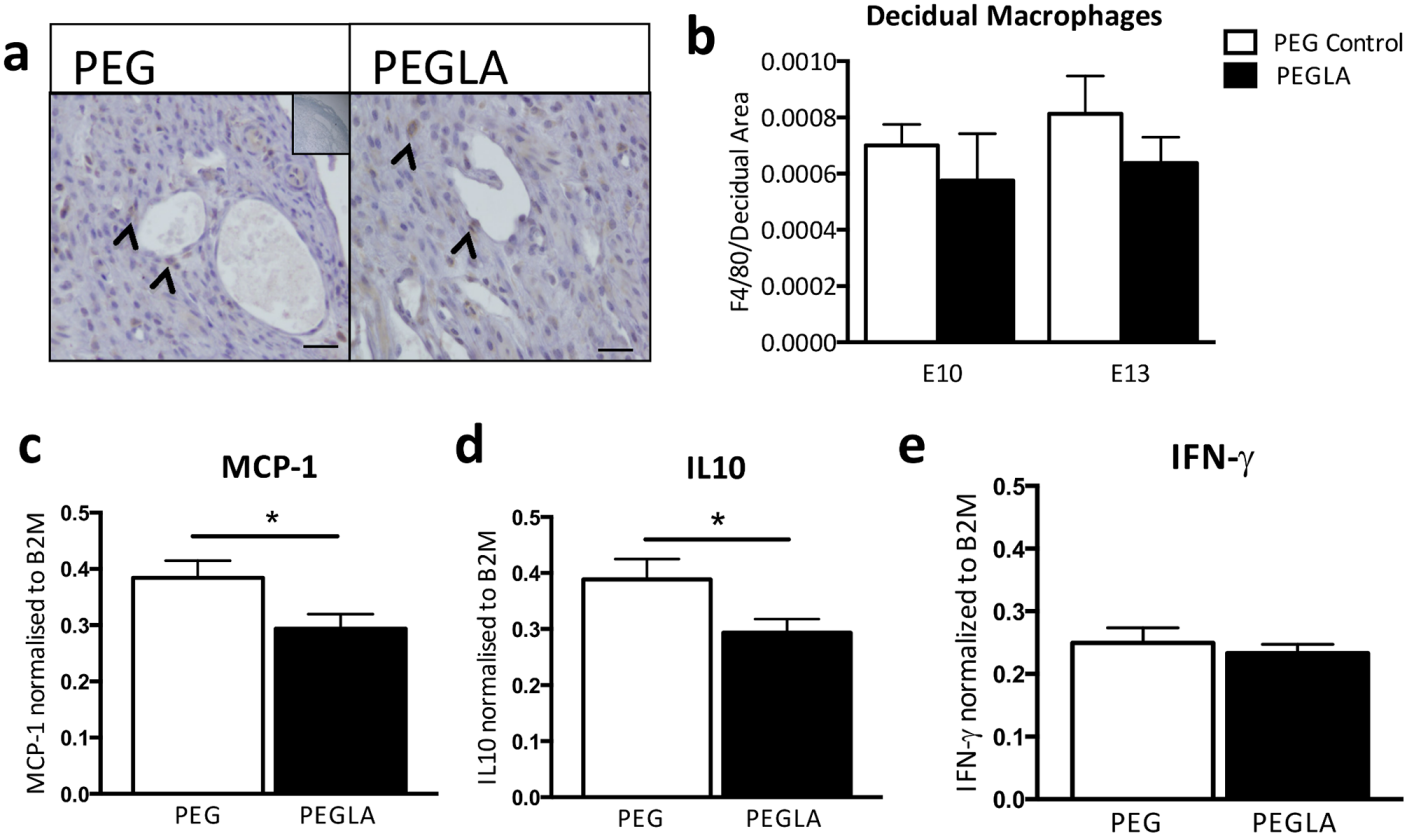
LIF inhibition during placental development alters decidual immune cell activation transcription factors. **Representative photomicrographs of** F4/80 macrophage immunostaining performed on E10 or E13 implantation sites treated with PEG control or PEGLA from E8-10 or E10-13 respectively **(a)**. Bars represent 200μm. Inset is the negative control. Arrows denote F4/80-positive immunostained marcrophages. **(b)** Staining intensity was analysed in 3 mid-sagital implantation site sections per mouse (n = 4/group) and staining area per decidual are calculated and averaged using CellSens software. Data are mean ± SEM. **(c)** MCP-1, **(d)** IL-10 and **(e)** IFN-γ mRNA expression was determined by semi-quantitative PCR normalized to β2-microglobulin in PEG or PEGLA treated mouse decidual tissue. Data are mean ± SEM, students t-test, *p<0.05.

To determine the effect of LIF inhibition during trophoblast invasion and placentation in mice, we immunolocalized macrophages in PEG or PEGLA treated mouse implantation sites at E10 and E13. There was a trend in reduced macrophage number in mice treated with PEGLA compared to PEG control, but no significant difference at either E10 or E13 (Fig 5). There was a significant reduction in *MCP-1* and *IL-10* mRNA in PEGLA-treated mouse decidua compared to PEG control at E13 (Fig 5), though levels of *IFN-γ* mRNA were unchanged between groups (Fig 5).

## Discussion

This study is the first to determine the role of LIF in mediating trophoblast invasion during placental development *in vivo*. We found that *LIF* and its signalling components, *LIFR* and *gp130* are expressed in the mouse decidua at the transcript level during the time of trophoblast invasion and spiral artery remodeling. Furthermore, LIF and LIFR both co-localized to invasive endovascular EVTs within the mid-gestation mouse decidua, supporting a potential functional role in EVT invasion that has previously been reported in humans [24, 25]. By temporally blocking LIF action during the initiation of trophoblast invasion and placentation in mice using a unique antagonist, PEGLA, we demonstrated that LIF is required for normal trophoblast invasion *in vivo*.

In women the high levels of LIF expression in first trimester decidua and the localization of its receptor on trophoblast cells suggests paracrine actions during placentation [22, 23]. Previously, we localized LIF to the mouse decidua post-implantation at E5 [32], however this is before the time of EVT invasion and spiral artery remodeling during mouse pregnancy, which occurs predominantly between E8-13 [33]. In the present study, we confirmed *LIF* and *LIFR* mRNA production in the mouse decidua during this time and also EVT production of LIF and LIFR. However, we observed no decidual alterations following LIF inhibition, likely due to the fact that mice were administered with PEGLA from E8 of gestation onwards, after the time of critical establishment of the maternal decidua in mice [31].

As hypothesized, LIF inhibition resulted in abnormalities in EVT invasion and spiral artery remodeling in mice. These *in vivo* findings strongly support previous *in vitro* findings in humans where LIF has been shown to promote JEG-3 choriocarcinoma cell line invasion [34] and also primary trophoblast invasion [24, 25]. Meanwhile, PEGLA has been demonstrated to inhibit this effect [25]. In women, abnormal trophoblast invasion and impaired spiral artery remodeling is associated with pregnancy complications such as placental insufficiency, intrauterine growth restriction (IUGR) and preeclampsia [35]. At term, placental LIF is elevated in women with preeclampsia [32]. However, it is unclear whether LIF may be a causal factor or a byproduct of abnormal placentation. To our knowledge, LIF levels in maternal serum have not been investigated during the first trimester of pregnancy, when abnormal trophoblast invasion occurs in women, prior to the onset of preeclamptic symptoms. This may give some indication of placental LIF levels and offer some insight as to whether reduced LIF levels are present in women with likely impaired spiral artery remodeling as seen in the PEGLA-treated mice.

*In vitro* studies suggest that LIF may exert direct effects on trophoblasts and/or decidual cells to facilitate EVT invasion. Previously, we reported that LIF alters the balance between matrix metalloproteinase (MMPs) and tissue inhibitors matrix metalloproteinase (TIMPs) required for decidual extracellular matrix (ECM) breakdown [36], in primary human EVTs [24]. Strong expression of *LIF* mRNA has been detected in decidual leukocytes in women [22], suggesting that LIF may also act to facilitate immune-cell mediated trophoblast invasion and spiral artery remodeling. LIF is a macrophage-derived cytokine [37] and furthermore, macrophages numbers are reduced by more than half in implantation sites during early pregnancy in LIF-null mice [38], suggesting that LIF acts as a chemo-attractant for these cells.

We investigated this mechanism of LIF-mediated trophoblast invasion in the present study. However, there were no differences in the number of macrophages in between PEGLA or PEG-treated mouse implantation sites at E10 or E13. Macrophages were detected by immunohistochemistry in multiple decidual cross-sections per mouse, although a preferable method from complete quantitation of these cells could be by flow cytometry. Our findings could also suggest that LIF is required for macrophage recruitment very early during pregnancy in mice, prior to E8 when PEGLA was administered. Indeed, changes in macrophage recruitment in LIF^-/-^ mice were found as early as post-implantation D3 [38]. Despite this finding, interestingly, we did see a reduction in transcript factors required for macrophage recruitment and activation, suggesting that LIF may contribute to macrophage activation status.

Decidual macrophages are the second most abundant immune cell population at the implantation site in women during the first trimester, comprising 20–30% of immune cells in the uterine decidua [39, 40]. Dysregulated macrophage activation in the decidua has been implicated in recurrent miscarriages [41]. Thus, understanding the signalling pathways that regulate decidual macrophages may elucidate causes of early pregnancy failures. Macrophages are broadly classified into the classically activated M1 phenotype or the alternatively activated M2 phenotype. Macrophages can be polarized by IFN-γ into M1, which are ‘pro-inflammatory’, present antigens, produce reactive oxygen species and skew cell mediated immune responses [26]. Alternatively, IL-10, among other factors can induce M2 polarization, which promote tissue remodeling and immune tolerance [26]. During pregnancy, MCP-1 (CCL2) produced by decidual cells attracts new macrophages into the decidua, leading to protection against rejection of the fetus and appropriate vessel remodeling by balancing the M1/M2 ratio [26]. MCP-1 transcript levels were significantly reduced in PEGLA-treated decidual tissue compared to control. Although there were no statistical differences in the numbers of decidual F4/80-positive macrophages between treatment groups, there was a trend in reduced macrophage numbers in PEGLA-treated decidua at both E10 and E13, which supports the reduced MCP-1 transcript levels, suggesting that reduced LIF signalling could impair macrophage recruitment. It would be interesting to determine the effect of impaired LIF signalling earlier in pregnancy during decidualization, when macrophage recruitment is maximal [42]. Furthermore, IL-10 transcript levels were significantly down regulated following LIF inhibition compared to control, suggesting that LIF may alter macrophage polarization. However, this would need to be investigated using specific macrophage markers to differentiate the subtypes.

## Conclusion

In this study we have demonstrated that targeted inhibition of LIF signalling during mid gestation leads to abnormal trophoblast invasion and maternal decidual spiral artery remodeling *in vivo* in mice. These data warrant future studies investigating the broader relationship between LIF and immune cells during pregnancy and strengthen the rationale that LIF is a critical player in the establishment of a healthy pregnancy.

## Supporting Information

S1 Table Arrive Checklist. This study was performed in compliance with the Animal Research: Reporting of *In Vivo* Experiments (ARRIVE). (PDF)
